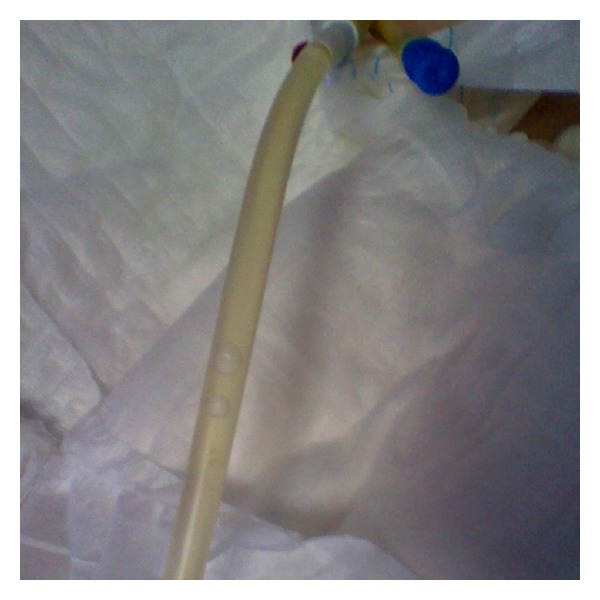# A PUBS Case in a Palliative Care Unit Experience

**DOI:** 10.1155/2014/169782

**Published:** 2014-09-15

**Authors:** M. R. Restuccia, M. Blasi

**Affiliations:** Palliative Care at Home Unit, Sue Ryder Foundation Onlus, Via della Rustica 218, 00155 Rome, Italy

## Abstract

Purple urine bag syndrome (PUBS) is a rare condition in which purple discoloration of the collecting bag and its associated tubing occurs. It is considered a benign condition. PUBS is usually associated with urinary tract infection occurring in elderly bedridden women, with chronic urinary catheterization. This syndrome is usually reported to occur in alkaline urine, but here we describe a rare case of PUBS involving acidic urine.

## 1. Introduction

Barlow and Dickson [1] first described a rare and fortunately harmless, but surprising, syndrome occurring in patients using permanent bladder catheters.

It became known as PUBS (Purple Urine Bag Syndrome). The majority of patients were females, bedridden, and with different comorbidities.

The distinctive trait of this syndrome concerns the discoloration of the bag and the duct while the urine inside remains untouched or, at most, appears muddy [1].

A remarkable bacterial count seems to be a key factor in revealing this syndrome, especially if there are* Proteus*,* Escherichia*, and* Pseudomonas* colonies. Another distinctive feature seems to be an alkaline pH but it rarely occurs in an acidic one [2].

It is believed that this discoloration is due to an indigo and indirubin mix, resulting from bacterial enzymes during tryptophan metabolism at different sites [3].

Below we describe a rare case of PUBS involving acidic urine.

## Case Report

During our experience in Palliative Care at home, we came across this curious case which, at first, struck us by being quite surprising.

The patient was 81-year-old female with prior gastric resection for advanced gastric cancer. There was a clinical history of chronic heart failure, chronic respiratory failure, hypertension, diabetes mellitus type II, multiple vertebral collapses, bedridden and bearer of chronic bladder catheter.

**Figure 1:**
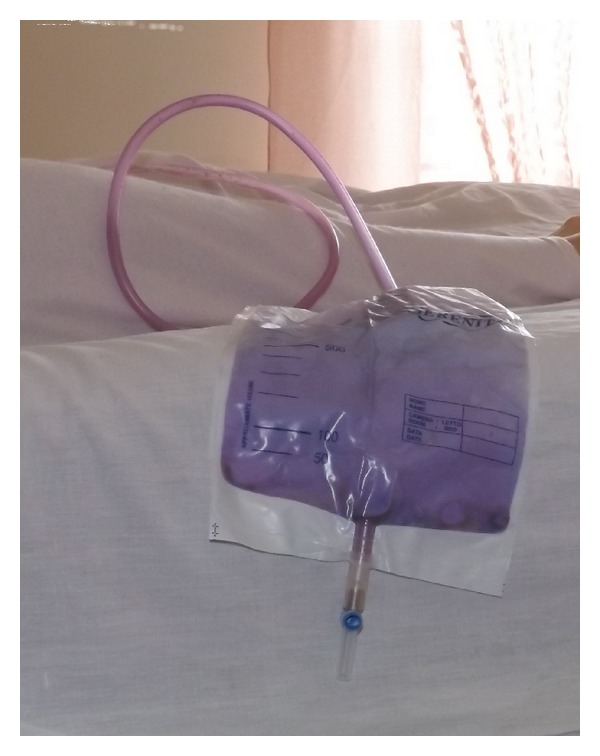


During one of the regular examinations, we noticed a strange discoloration of the bag and drainage tube of the catheter, while the urine inside the bag remained normal in color (Figure 1).

The patient appeared completely asymptomatic and denied recent episodes of fever, vomiting, or having taken drugs other than her usual home therapy or having changed her diet.

Her urine was then carefully analyzed with the results shown below:

- colonies of leukocytes (20–30 per field);
- mucus pus: abundant;
- haemoglobin ++;
- rich mixed microbial flora;
- acidic pH.

Following a urinary culture, abundant colonies of* Escherichia coli* (1.000.000) were isolated and proved to be sensitive to ciprofloxacin and aztreonam.

After antibiotic therapy the purple color disappeared (Figure 2).

## 3. Discussion

The situation described here, nowadays known as PUBS, is associated with females, alkaline urine, constipation, hospitalization, and use of plastic (PVC) urinary catheters [4].

All these factors, in combination with a high bacterial count in the urine and a diet rich in tryptophan, facilitate the development of this syndrome which is often associated with* Providencia* spp.,* E. coli*,* Proteus* spp.,* Pseudomonas* spp.,* Klebsiella pneumoniae*,* Morganella *spp., and* Enterococcus* spp.

Less commonly reported associations were with* Citrobacter* spp.,* Staphylococcus* spp., and* Streptococcus* spp. [5].

The pathogenesis of this syndrome seems to lie in tryptophan metabolism induced by intestinal bacteria. This metabolite is then converted to indoxyl sulphate in the liver and is excreted in urine. The Gram-negative bacteria, such as those mentioned above, and which possess an enzyme, indoxil phosphatase/sulphatase, act on indoxyl sulphate converting it to the indigo coloration. The purple discoloration of the bag occurs as a result of mixing indirubin (which is red) with fine indigo blue crystals over plastic.

Other authors, such as Barlow and Dickson, assume that indoxyl sulphate is oxidized to become indigo after urinary excretion and exposure to air. Vicuna and Lorenzo [6] support the theory that indicanuria in patients leads to a blue color only when urine is treated with an oxidizing agent such as sodium hypochlorite (bleaching agent) [6].

The main causes of PUBS have not been yet clarified and remain elusive.

## 4. Conclusion

Cases reported in literature together with our case demonstrate several important factors that contribute to PUBS. These cases appear to be related to urinary tract infections that resolved after antibiotic treatment. PUBS is a benign condition without any major consequences.

We believe that, nevertheless, clinicians should be aware of this syndrome in chronically catheterized patients. It indicates the presence of a urinary tract infection easily treatable, but proving to be fatal when left untreated in elderly patients with significant comorbidities.

## Figures and Tables

**Figure 2:**